# Cyc8p and Tup1p transcription regulators antagonistically regulate Flo11p expression and complexity of yeast colony biofilms

**DOI:** 10.1371/journal.pgen.1007495

**Published:** 2018-07-02

**Authors:** Phu Van Nguyen, Otakar Hlaváček, Jana Maršíková, Libuše Váchová, Zdena Palková

**Affiliations:** 1 Department of Genetics and Microbiology, Faculty of Science, Charles University, BIOCEV, Vestec, Czech Republic; 2 Institute of Microbiology of the Czech Academy of Sciences, BIOCEV, Vestec, Czech Republic; University College Dublin, IRELAND

## Abstract

Yeast biofilms are complex multicellular structures, in which the cells are well protected against drugs and other treatments and thus highly resistant to antifungal therapies. Colony biofilms represent an ideal system for studying molecular mechanisms and regulations involved in development and internal organization of biofilm structure as well as those that are involved in fungal domestication. We have identified here antagonistic functional interactions between transcriptional regulators Cyc8p and Tup1p that modulate the life-style of natural *S*. *cerevisiae* strains between biofilm and domesticated mode. Herein, strains with different levels of Cyc8p and Tup1p regulators were constructed, analyzed for processes involved in colony biofilm development and used in the identification of modes of regulation of Flo11p, a key adhesin in biofilm formation. Our data show that Tup1p and Cyc8p regulate biofilm formation in the opposite manner, being positive and negative regulators of colony complexity, cell-cell interaction and adhesion to surfaces. Notably, in-depth analysis of regulation of expression of Flo11p adhesin revealed that Cyc8p itself is the key repressor of *FLO11* expression, whereas Tup1p counteracts Cyc8p’s repressive function and, in addition, counters Flo11p degradation by an extracellular protease. Interestingly, the opposing actions of Tup1p and Cyc8p concern processes crucial to the biofilm mode of yeast multicellularity, whereas other multicellular processes such as cell flocculation are co-repressed by both regulators. This study provides insight into the mechanisms regulating complexity of the biofilm lifestyle of yeast grown on semisolid surfaces.

## Author summary

Yeast biofilms have become an increasingly important clinical problem over the years. Biofilms are often associated with infections resistant to antifungals, which are particularly prevalent in immunosuppressed patients. High resistance is mediated by cell reprogramming leading to the specific organization of internal biofilm structure and development of numerous protective mechanisms including extracellular matrix formation. Colony biofilms, with architecture and protective mechanisms similar to natural biofilms, represent an ideal model for studying molecular mechanisms and regulations behind biofilm development and organization. Here, we describe a new mechanism of antagonistic regulation of biofilm-specific processes and formation of complex colony biofilm structure by Cyc8p and Tup1p transcriptional regulators. Both these regulators are widespread and conserved among yeasts forming clinically important biofilms, including pathogenic yeasts of *Candida* spp. The identification of Tup1p as a positive regulator of biofilm formation makes Tup1p-orthologs in yeast pathogens potential targets for the design of new strategies of treatment of biofilm infections.

## Introduction

In nature, microorganisms preferentially live within multicellular communities such as different types of biofilms and colonies [1–5]. Yeast biofilms are complex uniquely organized structures, in which cells are protected from hostile environments, including antifungals, host immune systems, and starvation. Active multidrug resistance transporters and protective extracellular matrix that are produced by subpopulations of differentiated cells in colony biofilms formed by wild *Saccharomyces cerevisiae* strains [6], contribute to this protection. Despite the identification of various genes and processes (including chromosome reorganization) involved in formation of colony biofilm structure [1,7,8], many of these processes seem to be specific to particular wild strains. An exception is the Flo11p cell wall adhesin, a key protein involved in several developmental processes including cell adhesion [9] and the formation of colony biofilms [10], flor biofilms [11], and mats [12]. Deletion of *FLO11* results in the formation of smooth colonies in various non-isogenic wild strains isolated from different habitats [13] as well as in Σ1278-derived strains [10,14]. *FLO11* mRNA levels are elevated in colony biofilms and lowered after phenotypic switching called domestication, during which cells are reprogrammed to form smooth colonies similar to colonies of laboratory strains, in which key features of biofilm-life style are switched off [13,15]. The *FLO11* promoter extends to 3 kb and contains at least four upstream activation sequences and nine elements involved in repression [16]. Thus, *FLO11* gene expression integrates signals from diverse signaling cascades, including the Ras-cyclic AMP-dependent kinase, mitogen-activated protein kinase (which controls filamentous growth) and the main glucose repression pathways. These pathways positively or negatively regulate *FLO11* expression in accordance with growth stage and nutritional conditions [1,16–18]. The expression of *FLO11* is also controlled by epigenetic mechanisms, including histone deacetylation, chromatin remodeling, non-coding RNAs and prion formation [1,19–23].

The Cyc8p (Ssn6p)-Tup1p complex is mostly known to function as a transcriptional co-repressor that is conserved in eukaryotic organisms including mammals [24]. Four molecules of Tup1p in concert with one molecule of Cyc8p form this complex [25] with a tendency to oligomerize [26], which regulates hundreds of *S*. *cerevisiae* genes involved in diverse pathways, such as glucose, starch and oxygen utilization, the response to osmotic stress, DNA repair, mating, sporulation, meiosis and flocculation [24,27,28]. Cyc8p-Tup1p does not bind directly to DNA but is brought to promoters *via* interactions with sequence-specific regulatory binding proteins, which coordinate the expression of specific subsets of genes [24]. Some data indicate that Cyc8p may play a more direct role in the repression [29]. Cyc8p-Tup1p can interact with Mig1p and Nrg1p, which bind to the promoters of glucose-repressed genes, such as *FLO11*, in the presence of glucose [17,30]. Cyc8p-Tup1p can also act as a transcriptional co-activator of various genes such as *HAP1* [31,32], *FRE2* [33], *ARG1* and *ARG4* in cooperation with Gcn4p [34], *TAT1* and *TAT2* [35], and genes induced by Hog1p in cooperation with Sko1p [36]. Genome-wide profiling of changes in nucleosome organization and gene expression that occur following the loss of *CYC8* or *TUP1* in *S*. *cerevisiae* laboratory strains show significant overlap, but additional changes result from the absence of either *TUP1* or *CYC8* [37]. Thus, the major function of Cyc8p and Tup1p in *S*. *cerevisiae*, identified so far, is the repression of pleiotropic gene targets mostly in the form of the Cyc8p-Tup1p co-repressor complex. In addition, several mutually independent repressor functions of Tup1p and Ssn6p (a functional homologue of *S*. *cerevisiae* Cyc8p) have been reported in *Candida albicans*, in which filamentous growth and hypha-specific genes are repressed by Tup1p independently of Ssn6p, whereas Ssn6p may act as a repressor of phenotypic switching independently of Tup1p [38]. Ssn6p was recently identified as a negative regulator of the opaque cell transcription program in white *C*. *albicans* cells and of the white cell transcription program in opaque cells [39]. Tup1p was reported to be a repressor of the opaque state and, together with its negative regulator Wor1p, has been proposed to control the opaque switch under different circumstances [40]. In addition to its interaction with Tup1p, Ssn6p interaction with histone deacetylase Rpd31p has been reported in *C*. *albicans* [41]. In this study, Ssn6p appeared to be a repressor of filamentation as well as of wrinkled colony morphology under particular conditions, independently of Tup1p, and some of these repressive effects were enhanced by deletion of *RPD31*. MoTUP1 was recently identified in *Magnaporthe oryzae* (a rice pathogen), and its deletion causes decreased pathogenicity of the fungus [42]. These studies suggest that Tup1p and Cyc8p play important roles in the pathogenicity of different fungi and that, in addition, these factors could have independent roles.

In this study, we provide clear evidence of the functions of the Tup1p and Cyc8p regulators in biofilm colony formation. We present evidence that Cyc8p itself is a repressor of *FLO11* gene expression and of the formation of the structured architecture of colony biofilms, whereas Tup1p counteracts Cyc8p, being a positive regulator of *FLO11* expression and colony complexity. Furthermore, we show that Tup1p regulates Flo11p accumulation at two different levels—gene expression and Flo11p stability. In addition to Flo11p, other features that are important for colony biofilm formation, such as cell invasiveness, adhesion to solid surfaces and presence of fibers connecting the cells, are also antagonistically regulated by Cyc8p and Tup1p. Conversely, features that are related to other types of multicellularity, such as cell flocculation, are co-repressed by both regulators.

## Results and discussion

### Cyc8p and Tup1p exhibit antagonistic effects on the architecture of colony biofilms

A series of strains was constructed producing different levels of Cyc8p and Tup1p regulators (Table 1) derived from the parental BR-F strain (wt strain; [43]), which forms structured colony biofilms [6]. The *tup1* strain (*tup1*/*tup1*) was prepared by deleting both alleles of *TUP1*, but we did not succeed in preparing a *cyc8* strain (*cyc8*/*cyc8*). As the *CYC8* gene is essential in the Σ1278 strain-background [44], resembling in several aspects wild yeast strains, this gene may also be essential in the BR-F strain. Therefore, we constructed strain p_GAL_-*CYC8* (*cyc8*/p_GAL_-*CYC8*), in which one *CYC8* allele is deleted and the second placed under the control of the *GAL1*-inducible promoter (p_GAL_), which provides only very low (basal) level of *CYC8* expression in the absence of galactose. The decreased level of *CYC8* mRNA and level of Cyc8p in this p_GAL_-*CYC8* strain (grown without galactose), compared with the BR-F strain, was confirmed by northern blot (S1A Fig) and LC-MS/MS (see below), respectively. We also prepared strain p_TEF_-*CYC8* (*CYC8*/p_TEF_-*CYC8*) constitutively over-expressing *CYC8* from the *TEF1* promoter (p_TEF_).

**Table 1:** Yeast strains.

Name	Genotype	Colony morphology*	Source
BR-F	*MATa*/*MATα*	structured	[43]
BR-F-*flo11*	*MATa/MATα flo11Δ*::*kanMX/flo11Δ*::*ble*	smooth	[6]
*tup1*	*MATa*/*MATα*, *tup1Δ*::*KanMX*, *tup1Δ*::*nat1*	smooth	this study
p_TEF_-*CYC8*	*MATa*/*MATα*, *nat1-TEF1-CYC8/CYC8*	smooth	this study
p_GAL_-*CYC8*	*MATa*/*MATα*, *cyc8Δ*::*KanMX*, *nat1-GAL1-CYC8*	structured**	this study
p_TEF_-*TUP1*	*MATa*/*MATα*, *nat1-TEF1-TUP1/TUP1*	structured	this study
p_GAL_-*TUP1*	*MATa*/*MATα*, *tup1Δ*::*KanMX*, *nat1-GAL1-TUP1*	smooth**	this study
BR-F-Flo11p-GFP	*MATa*/*MATα*, *FLO11-GFP/FLO11*	structured	[15]
Flo11p-GFP/p_TEF_-*CYC8*	*MATa*/*MATα*, *FLO11-GFP/FLO11*, *nat1-TEF1-CYC8/CYC8*	smooth	this study
Flo11p-GFP/p_TEF_-*TUP1*	*MATa*/*MATα*, *FLO11-GFP/FLO11*, *nat1-TEF1-TUP1/TUP1*	structured	this study
Flo11p-GFP/p_GAL_-*CYC8*	*MATa*/*MATα*, *FLO11-GFP/FLO11*, *cyc8Δ*::*KanMX*, *nat1-GAL1-CYC8*	structured**	this study
Flo11p-GFP/p_GAL_-*TUP1*	*MATa*/*MATα*, *FLO11-GFP/FLO11*, *tup1Δ*::*KanMX*, *nat1-GAL1-TUP1*	smooth**	this study
*sfl1*/*CYC8*/p_TEF_-*CYC8*	*MATa*/*MATα*, *FLO11-GFP/FLO11*, *nat1-TEF1-CYC8/CYC8*, *sfl1Δ*::*KanMX*, *sfl1Δ*::*HygR*	smooth	this study
*nrg1*/*CYC8*/p_TEF_-*CYC8*	*MATa*/*MATα*, *FLO11-GFP/FLO11*, *nat1-TEF1-CYC8/CYC8*, *nrg1Δ*::*KanMX*, *nrg1Δ*::*HygR*	smooth	this study
*mig1*/*CYC8*/p_TEF_-*CYC8*	*MATa*/*MATα*, *FLO11-GFP/FLO11*, *nat1-TEF1-CYC8/CYC8*, *mig1Δ*::*KanMX*, *mig1Δ*::*HygR*	smooth	this study
p_GAL_-*TUP1/*p_CUP_-*CYC8*	*MATa*/*MATα*, *tup1Δ*::*loxP*, *cyc8Δ*::*loxP*, *nat1-GAL1-TUP1*, *KanMX-CUP1-CYC8*	smooth**	this study
p_CUP_-*TUP1/*p_GAL_-*CYC8*	*MATa*/*MATα*, *tup1Δ*::*loxP*, *cyc8Δ*::*loxP*, *KanMX-CUP1-TUP1*, *nat1-GAL1-CYC8*	smooth**	this study
Flo11p-GFP/p_GAL_-*TUP1/* p_CUP_-*CYC8*	*MATa*/*MATα*, *FLO11-GFP/FLO11*, *tup1Δ*::*loxP*, *cyc8Δ*::*loxP*, *nat1-GAL1-TUP1*, *KanMX-CUP1-CYC8*	smooth**	this study
Flo11p-GFP/p_CUP_-*TUP1/* p_GAL_-*CYC8*	*MATa*/*MATα*, *FLO11-GFP/FLO11*, *tup1Δ*::*loxP*, *cyc8Δ*::*loxP*, *KanMX-CUP1-TUP1*, *nat1-GAL1-CYC8*	smooth** ^$^	this study

*For colonies grown on GMA medium

** Without induction

$ Semi-structured when medium contains traces of Cu^2+^

**Fig 1:**
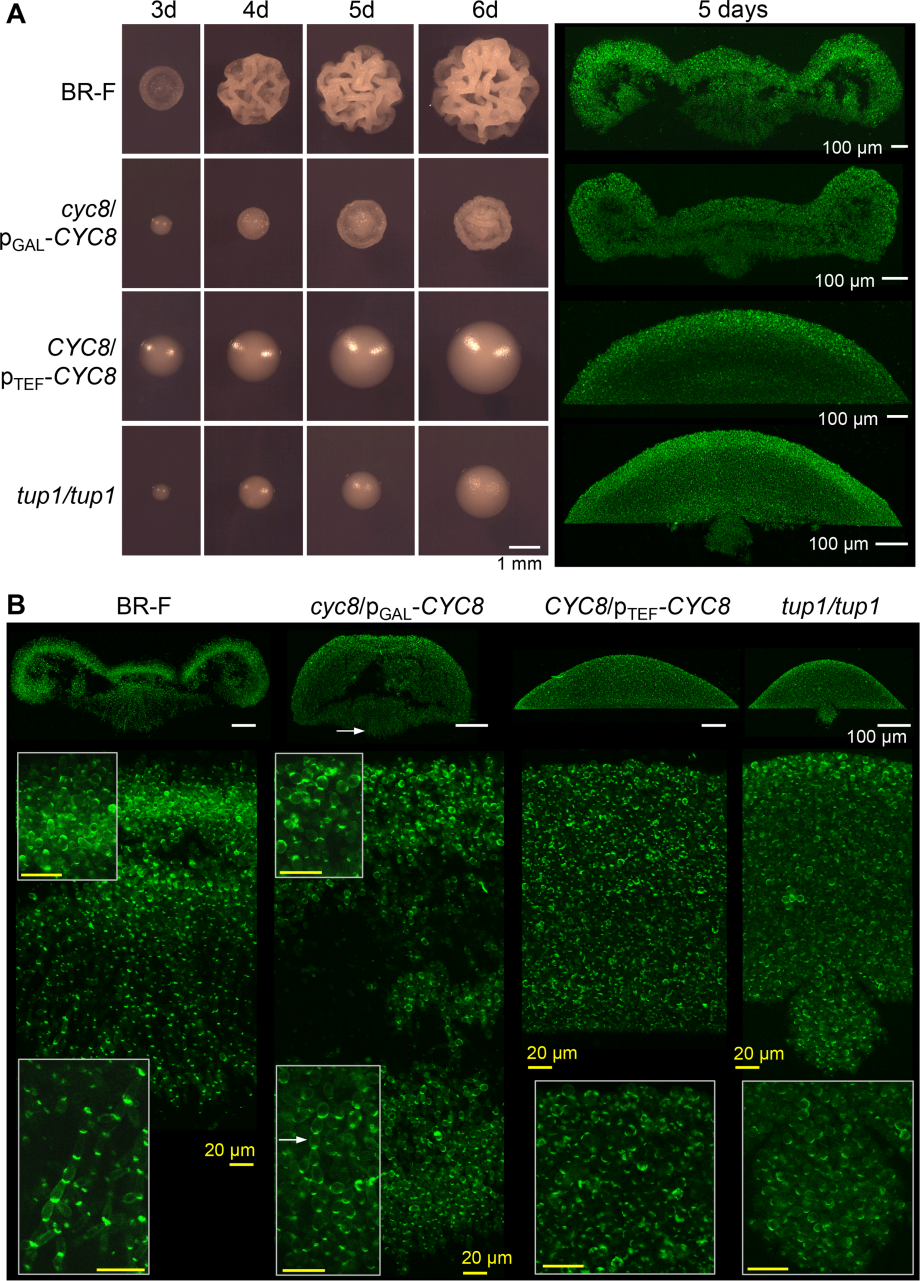
Development and architecture of colonies with altered levels of Cyc8p and Tup1p. A, Development of colonies grown on GMA at a density of ~10^3^ colonies per plate (left panel). Two photon excitation confocal microscopy (2PE-CM) of colony cross-sections stained with Calcofluor white (false green color); colonies were grown for 5 days on GMA at a density of ~3 x 10^3^ colonies per plate (right panel). B, 2PE-CM of colony cross-sections stained with Calcofluor white (false green color). Colonies were grown for 3 days on GMA at a density of ~3 x 10^3^ colonies per plate. Upper part, whole colonies (20x objective); lower part, central colony parts shown at a higher magnification (63x objective), insets: details of aerial and subsurface cells (strains BR-F and p_GAL_-*CYC8*); detail of central part (p_TEF_-*CYC8*); detail of the colony bottom (*tup1*). White bar, 100 μm; yellow bar, 20 μm. Arrow indicates chains of rounded cells invading the agar.

Unexpectedly, deletion of *TUP1* and *CYC8* over-expression resulted in a similar, very prominent change in colony architecture indicating opposing roles of Tup1p and Cyc8p in biofilm formation (Fig 1A). In both cases, the strains formed smooth colonies. Conversely, although reduced *CYC8* expression slowed the growth of the p_GAL_-*CYC8* strain, this strain formed structured colony biofilms that gradually developed morphology similar to that of wt strain biofilms (S1B Fig). Hence, 3-day-old p_GAL_-*CYC8* colonies exhibited an architecture (Fig 1B) with features typical of younger (40-h-old) structured biofilms formed by the wt strain and 5-day-old p_GAL_-*CYC8* colony biofilms resemble 3-day-old biofilms of the wt strain (S1B Fig and [6]).

### Cyc8p and Tup1p regulate the production of Flo11p adhesin in an opposite manner

**Fig 2:**
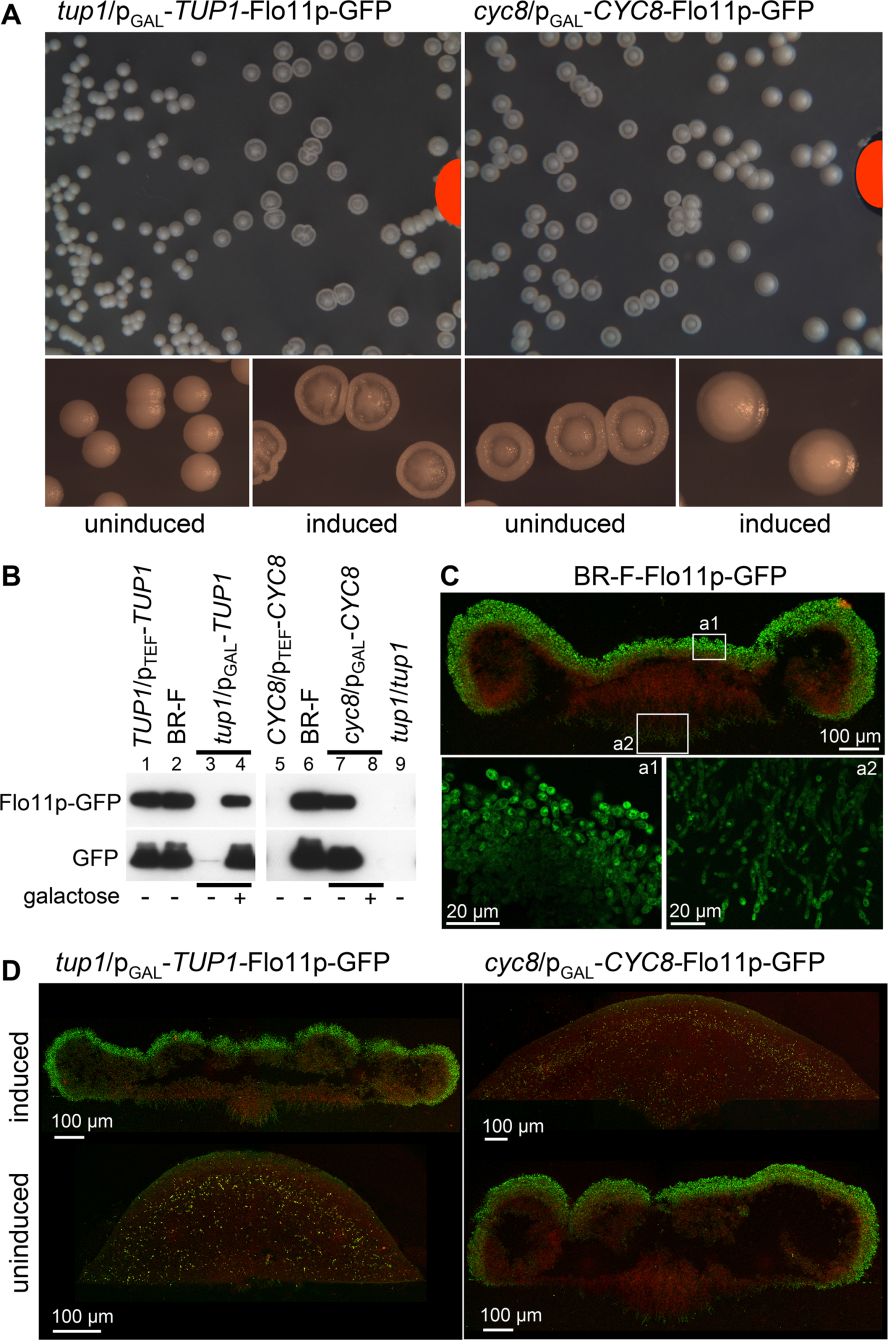
Expression of Flo11p adhesin in colonies with altered levels of Cyc8p and Tup1p. A, Effects of *TUP1* (left) and *CYC8* (right) expression induced by galactose diffusing from the wells in the agar (marked in red) on colony morphology. 120 μl of 10% galactose was applied to each well. Morphologies of wt colonies treated in the same way are shown in S5 Fig. B, Flo11p-GFP in 3-day-old colonies of Flo11p-GFP strains grown on GMA. Representative result of the 4 experiments is shown. Western blot loading controls are in S4 Fig. C, 2PE-CM of cross-sections of 3-day-old colonies of BR-F-Flo11p-GFP strain grown on GMA. Green, Flo11p-GFP; red, cell autofluorescence (in upper picture). Plating density ~4–6 x 10^3^ colonies per plate. D, 2PE-CM of cross-sections of colonies from the plates with galactose induction (panel A). Induced colonies localized close to the galactose source and uninduced colonies localized to the plate margin are shown. Green, Flo11p-GFP; red, cell autofluorescence. Plating density ~4–6 x 10^3^ colonies per plate.

Flo11p is essential for colony biofilm formation. Therefore, we investigated the potential role of Tup1p and Cyc8p in Flo11p expression. We prepared strains p_GAL_-*CYC8*-Flo11p-GFP (*cyc8*/p_GAL_-*CYC8*-Flo11p-GFP) and p_GAL_-*TUP1*-Flo11p-GFP (*tup1*/p_GAL_-*TUP1*-Flo11p-GFP) (derived from the BR-F-Flo11p-GFP strain; [15]), in which *CYC8* and *TUP1* expression is inducible by galactose, to monitor Flo11p-GFP levels in the context of colony biofilm morphology. Presence of galactose in GMA partially affects the colony appearance, slightly reducing the structured morphology, as has similarly been shown for glucose YEPD medium [45]. p_GAL_-*TUP1*-Flo11p-GFP and p_GAL_-*CYC8-*Flo11p-GFP colonies were, therefore, first grown on GMA plates without galactose for 3 days and then expression of p_GAL_-controlled genes was induced for ~18 h by adding galactose to wells in the agar. Fig 2A shows that in areas of higher galactose concentration (near the wells), colony morphologies changed due to the induction of *TUP1* (smooth → structured) or *CYC8* (structured → smooth), whereas colonies located far from the galactose source retained their original morphologies. Western blots showed that Flo11p-GFP is produced in high levels in p_GAL_-*TUP1*-Flo11p-GFP colonies induced by galactose (Fig 2B, lane 4), whereas Flo11p-GFP production was totally abolished when *CYC8* over-expression was induced by galactose in p_GAL_-*CYC8*-Flo11p-GFP colonies (lane 8). In accordance, Flo11p-GFP was undetectable in p_TEF_*-CYC8-*Flo11p-GFP (*CYC8*/p_TEF_-*CYC8-*Flo11p-GFP*)* colonies constitutively overexpressing *CYC8* (lane 5), whereas Flo11p-GFP level in p_TEF_*-TUP1*-Flo11p-GFP (*TUP1*/p_TEF_-*TUP1-*Flo11p-GFP) (lane 1) colonies was similar to that of wt colonies (lane 2). Two photon excitation confocal microscopy (2PE-CM) showed that in 3-day-old wt colonies, Flo11p-GFP is present at higher levels in cells at the aerial surface of wt colonies and in cells forming the tips of “roots” invading the agar (Fig 2C). A similar pattern of Flo11p-GFP was observed in structured p_GAL_-*TUP1*-Flo11p-GFP colonies near the galactose source and in structured p_GAL_-*CYC8*-Flo11p-GFP colonies that were localized far from the galactose source and were thus not induced (Fig 2D). However, Flo11p-GFP was hardly detectable in smooth colonies of both strains.

**Fig 3:**
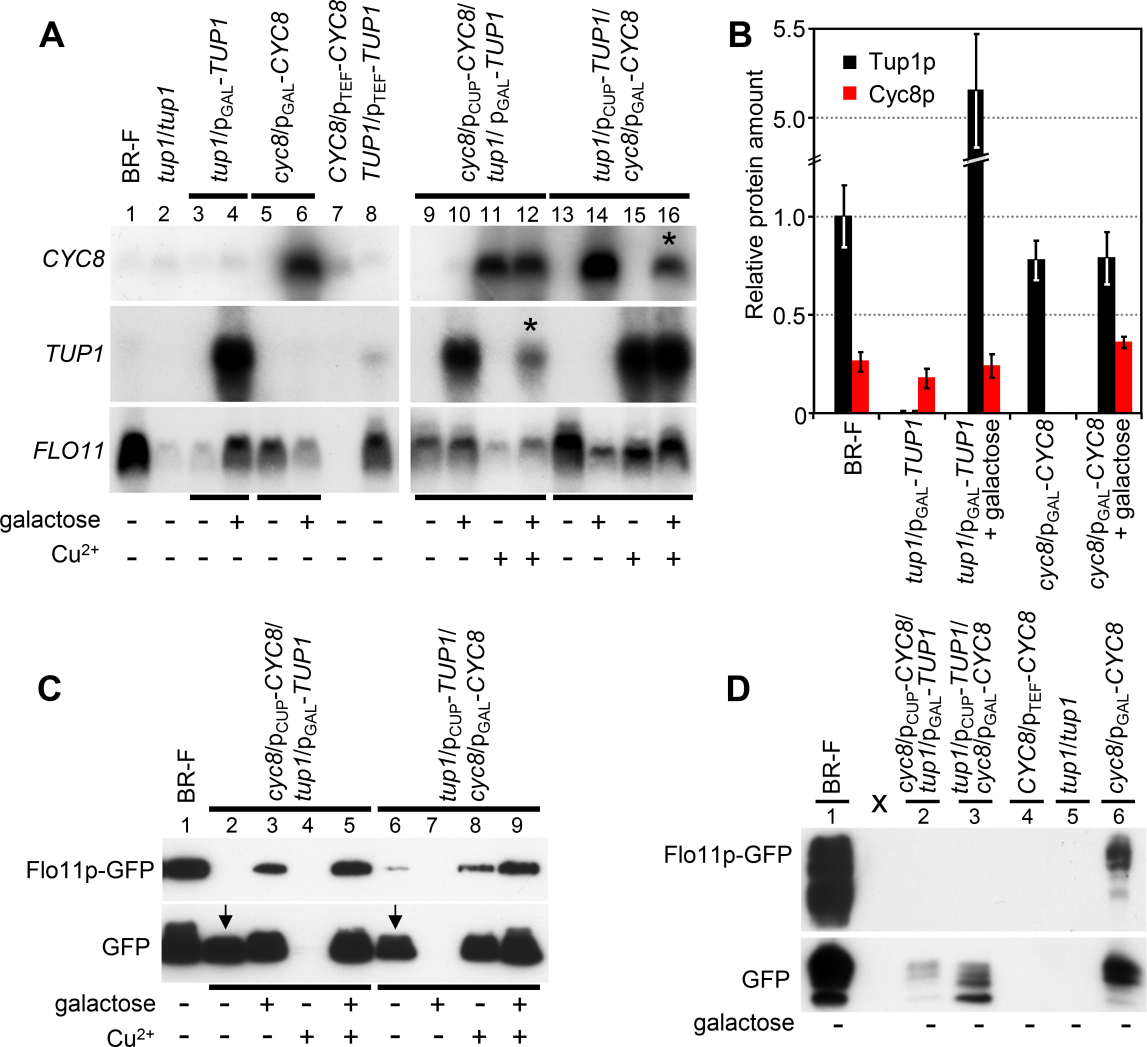
Levels of *CYC8*, *TUP1* and *FLO11* mRNAs and respective proteins in strains with differently altered levels of Cyc8p and Tup1p regulators. A. Northern blot of *CYC8*, *TUP1* and *FLO11* mRNAs from 3-day-old colonies grown on GMA and then induced/uninduced 4 h with galactose and/or copper. Representative result of the 4 experiments is shown. Northern blot loading controls are in S4 Fig. Asterisks indicate reduction of transcription from p_GAL_ promoter in the presence of copper. B. Levels of Tup1p and Cyc8p quantified by LC-MS/MS. Colonies were grown 3 days on GMA and then were induced/uninduced by galactose for 4 h. Protein amounts were related to the amount of Tup1p in BR-F colonies. C. Levels of Flo11p-GFP and free GFP in lysates from 3-day-old colonies (prepared from whole colony biomass including extracellular material) of respective strains grown on GMA, induced/uninduced for 18 h by galactose and/or copper. Arrows points to total degradation of Flo11p-GFP to GFP in particular samples. D. Levels of Flo11p-GFP and free GFP in the extracellular fluid extracted from 50 mg of wet weight biomass of 3-day-old colonies grown on GMA without galactose or copper induction.

To further clarify regulatory functions of Tup1p and Cyc8p, we compared amounts of *TUP1* and *CYC8* mRNAs and proteins in wt colonies and colonies of above described strains with differently manipulated levels of Cyc8p or Tup1p (Fig 3A, left part, and 3B). Colonies were grown for 3 days on GMA and then induced by galactose (or treated with distilled water as a control) for 4 hours. This induction greatly increased *TUP1* and *CYC8* mRNA levels, respectively, in p_GAL_-*TUP1* and p_GAL_-*CYC8* strains (Fig 3A, lanes 4 and 6, respectively). Conversely, amounts of both *CYC8* and *TUP1* mRNAs were only slightly increased, as compared with mRNA levels in wt colonies, when expression was controlled by the moderate, constitutive p_TEF_ promoter (lanes 7 and 8). Labeling of both Tup1p and Cyc8p proteins with GFP or 6HA tags resulted in dysfunctional proteins and commercial anti-Tup1p and anti-Cyc8p primary antibodies generated high unspecific background. Therefore, we quantified Tup1p and Cyc8p approximate concentrations in cells from 3-day-old colonies induced/non-induced by galactose for 4 hours by label free LC-MS/MS (Fig 3B). Contrary to *TUP1* and *CYC8* mRNA levels, which both were highly elevated when expression was induced by galactose (Fig 3A, lanes 4 and 6), differing enhancement of Cyc8p and Tup1p protein concentration was observed in p_GAL_-*CYC8* and p_GAL_-*TUP1* 3-day-old colonies. Whereas Cyc8p level increased only by 40% (~1.4 times), Tup1p level increased more than 5 times as compared with wt colonies (Fig 3B). In accordance with mRNA analysis (Fig 3A, lines 5 and 3), neither Cyc8p nor Tup1p were detected without galactose induction in p_GAL_-*CYC8* and p_GAL_-*TUP1* colonies, respectively (Fig 3B).

Analyses of *FLO11* mRNA levels (Fig 3A) showed that Cyc8p and Tup1p affect *FLO11* gene expression in opposite ways and with differing efficiencies. *CYC8* constitutive overexpression in p_TEF_-*CYC8* colonies resulted in absence of *FLO11* mRNA (Fig 3A, lane 7) and of Flo11p-GFP protein (Fig 2B, lane 5), thus confirming Cyc8p as a *FLO11* gene repressor. *TUP1* deletion in the presence of functional Cyc8p (*tup1* strain) resulted in the absence of Flo11p (Fig 2B, lane 9), but a small amount of *FLO11* mRNA was still detectable (Fig 3A, lane 2). This result confirmed that *FLO11* transcription is enhanced by Tup1p, but when the *TUP1* gene was deleted, some transcription of *FLO11* still occurred. In accordance, the level of *FLO11* mRNA was significantly reduced after 4 h of galactose induction of p_GAL_-*CYC8* colonies and the level of *FLO11* mRNA was significantly increased after 4 h of induction of p_GAL_-*TUP1* colonies (Fig 3A, lane 6 and 4, respectively). 18 h after galactose induction, Flo11p protein levels increased from non-detectable to a level comparable with the wt strain in p_GAL_-*TUP1* colonies (Fig 2B, compare lanes 3 and 4; Fig 2D) and decreased from a wt-like to non-detectable level in p_GAL_-*CYC8* colonies (Fig 2B, compare lanes 7 and 8; Fig 2D).

**Fig 4:**
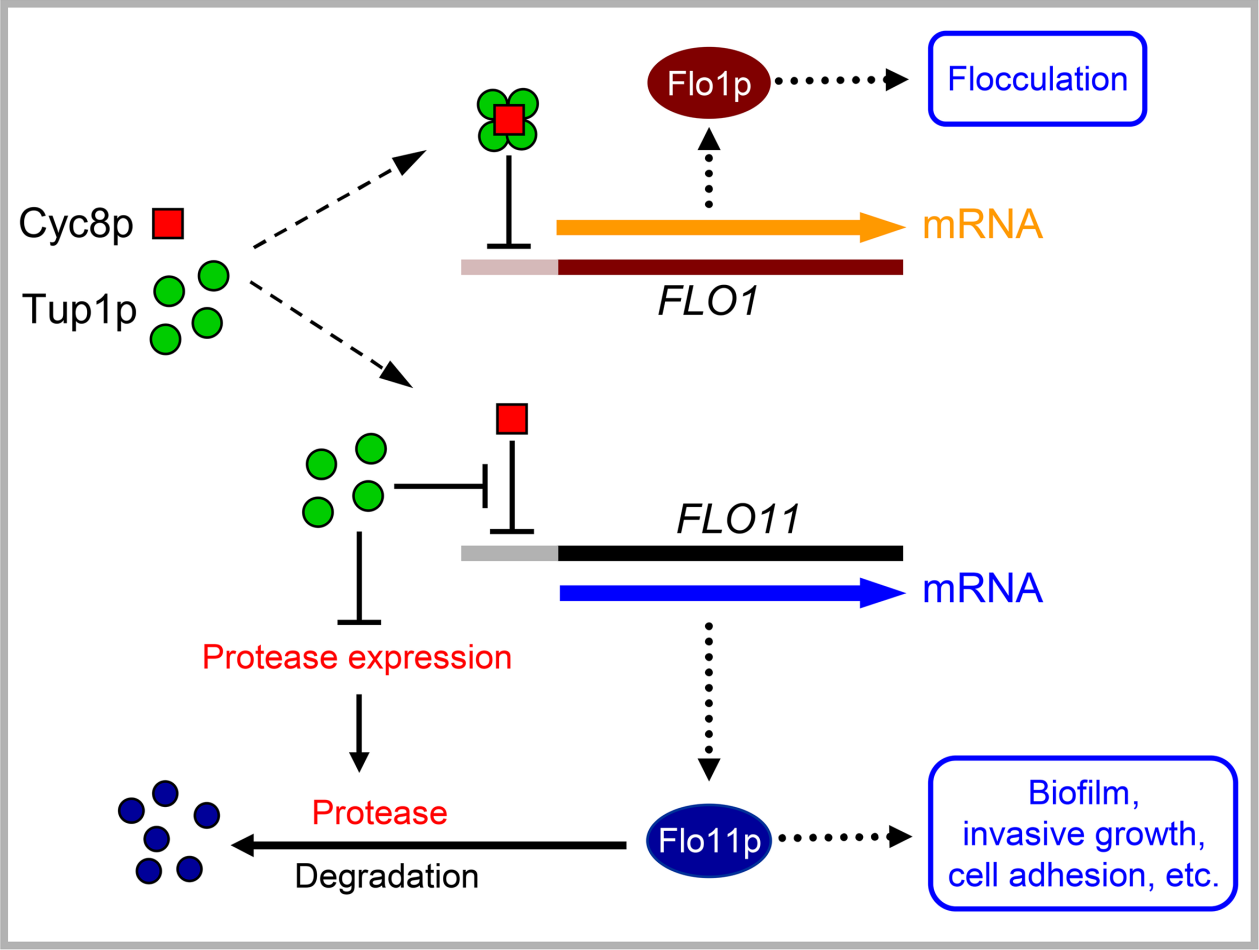
Model schematic of the regulatory functions of Cyc8p and Tup1p. In colony biofilms, levels of Cyc8p and Tup1p are balanced in such a way that Tup1p inhibits Cyc8p repressor (by forming Cyc8p-Tup1p complex), thus preventing *FLO11* repression. In addition, free Tup1p contributes to repression of putative extracellular protease that degrades Flo11p. The Cyc8p-Tup1p complex represses other cellular functions, such as cell flocculation.

To further examine mutual effects of both regulators, we constructed an additional set of strains derived from the BR-F and BR-F-Flo11p-GFP strains, in which amounts of both regulators were adjustable by the inducing compound (galactose or copper) (Table 1, last four strains). We then evaluated levels of *TUP1*, *CYC8* and *FLO11* gene expression (mRNA) and levels of respective proteins. Results of *CYC8* and *TUP1* mRNA analysis after 4 h of galactose and/or copper induction of colonies of strains p_GAL_-*CYC8*/p_CUP_-*TUP1* (*cyc8*/p_GAL_-*CYC8*/*tup1*/p_CUP_-*TUP1*) and p_GAL_-*TUP1*/p_CUP_-*CYC8 (tup1*/p_GAL_-*TUP1*/*cyc8*/p_CUP_-*CYC8*) (Fig 3A, lanes 9–16) were consistent with results of induction experiments performed with strains, in which expression of only one of the regulators was adjustable and the second was controlled by its native promoter (Fig 3A, lanes 3–6). Only the level of p_GAL_-regulated mRNA (of both *TUP1* and *CYC8*) was partially diminished when copper was also present during galactose induction (Fig 3A; compare lanes 14 and 16 for *CYC8* and lanes 10 and 12 for *TUP1*, decreased level of mRNA is marked by asterisk). Since p_GAL_-regulated expression of *CYC8* and *TUP1* was also diminished by copper in p_GAL_-*CYC8* and p_GAL_-*TUP1* colonies, respectively (S2 Fig), copper seem to partially reduce transcription from the p_GAL_ promoter. Consistently with p_GAL_-*CYC8* and p_GAL_-*TUP1* induction experiments, increased level of Cyc8p caused a decrease in *FLO11* mRNA (Fig 3A, lanes 11 and 14) and in Flo11p concentrations (Fig 3C, lanes 4 and 7) in p_GAL_-*CYC8*/p_CUP_-*TUP1* and p_GAL_-*TUP1*/p_CUP_-*CYC8* colonies. However, the basal *FLO11* mRNA level when neither Cyc8p nor Tup1p was induced (Fig 3A, lanes 9 and 13) was higher than under conditions where a wt-level of Cyc8p was present (*tup1* or p_GAL_-*TUP1* colonies without galactose, Fig 3A, lanes 2 and 3). 4 h-induction of Tup1p by either inducing compound did not significantly increase the *FLO11* mRNA level (Fig 3A, lanes 10 and 15) above the basal level identified in the absence of both inducing compounds (lanes 9 and 13). In fact this basal level was lower in the p_CUP_-*CYC8*/p_GAL_-*TUP1* strain than in p_GAL_-*CYC8*/p_CUP_-*TUP1* strain (Fig 3A, compare lanes 9 and 13), possibly because of traces of copper in the medium which can slightly increase *CYC8* expression and thus the amount of Cyc8p repressor from the onset of colony growth. Altogether, these data further confirmed that Cyc8p is the main repressor of expression of the *FLO11* gene and indicated that Tup1p modulates the level of Cyc8p repressor, potentially via formation of Tup1p-Cyc8p complex (Fig 4). Analysis of Flo11p-GFP protein levels however suggested an additional function of Tup1p. Flo11p-GFP full length protein was almost undetectable in the absence of both regulators, whereas a high level of free GFP was present in these samples indicating that Flo11p-GFP synthesis was relatively high (in accordance with high basal level of *FLO11* mRNA, Fig 3A, lane 9 and 13), but that the protein was efficiently degraded (Fig 3C, lanes 2 and 6, arrows mark free GFP). These data indicate dual roles of Tup1p in regulation of Flo11p concentration in colonies and thus in regulation of colony biofilm complexity: counteracting *CYC8* repression of *FLO11* gene expression and preventing Flo11p degradation, possibly by repressing expression of a specific protease.

Flo11p is associated with the cell wall and it is partially shed from cells into the extracellular space [46]. We therefore examined further whether extracellular Flo11p-GFP is degraded and whether presence of Tup1p influences such degradation. As expected, neither Flo11p-GFP nor free GFP was detected in extracellular fluid from colonies of p_TEF_-*CYC8* and *tup1* strains (Fig 3D, lanes 4 and 5), in which *FLO11* expression is repressed by Cyc8p. In extracellular fluid from biofilm colonies of wt strain and p_GAL_-*CYC*8 strain without galactose, both partially degraded Flo11p-GFP and high level of free GFP were detected (Fig 3D, lanes 1 and 6), indicating degradation of a fraction of Flo11p-GFP, perhaps during its shedding. In colonies of both p_GAL_-*TUP1/*p_CUP_-*CYC8* and p_CUP_-*TUP1/*p_GAL_-*CYC8* strains with high basal levels of *FLO11* gene expression and protein production in the absence of both inducing compounds, free GFP only was present in extracellular fluid (Fig 3D, lanes 2 and 3). Consistently, colonies of these strains are smooth. These data indicate that Tup1p prevents degradation of extracellular Flo11p-GFP possibly via repression of expression of a cell wall associated or extracellularly localized protease. Differences in Flo11p processing (at several positions within the protein) were found in a strain defective in the kexin Kex2p [46], serine protease which cleaves precursors of secreted proteins in the trans-Golgi network. However, Flo11p was not identified in the screen of possible Kex2p substrates and does not contain prominent Kex2p cleavage sites (Lys-Arg at P1 and P2 position) [47]. Hence, Flo11p is probably not a direct target of Kex2p, but it could be cleaved by another secreted protease, the secretion and/or processing of which requires Kex2p.

### Cyc8p and Tup1p antagonistically regulate other colony biofilm specific processes

Next, we examined Cyc8p and Tup1p roles in regulation of other processes that are specific to colony biofilms, such as cell-substrate adhesion and agar penetration and cell-cell interaction via cell wall fibers.

**Fig 5:**
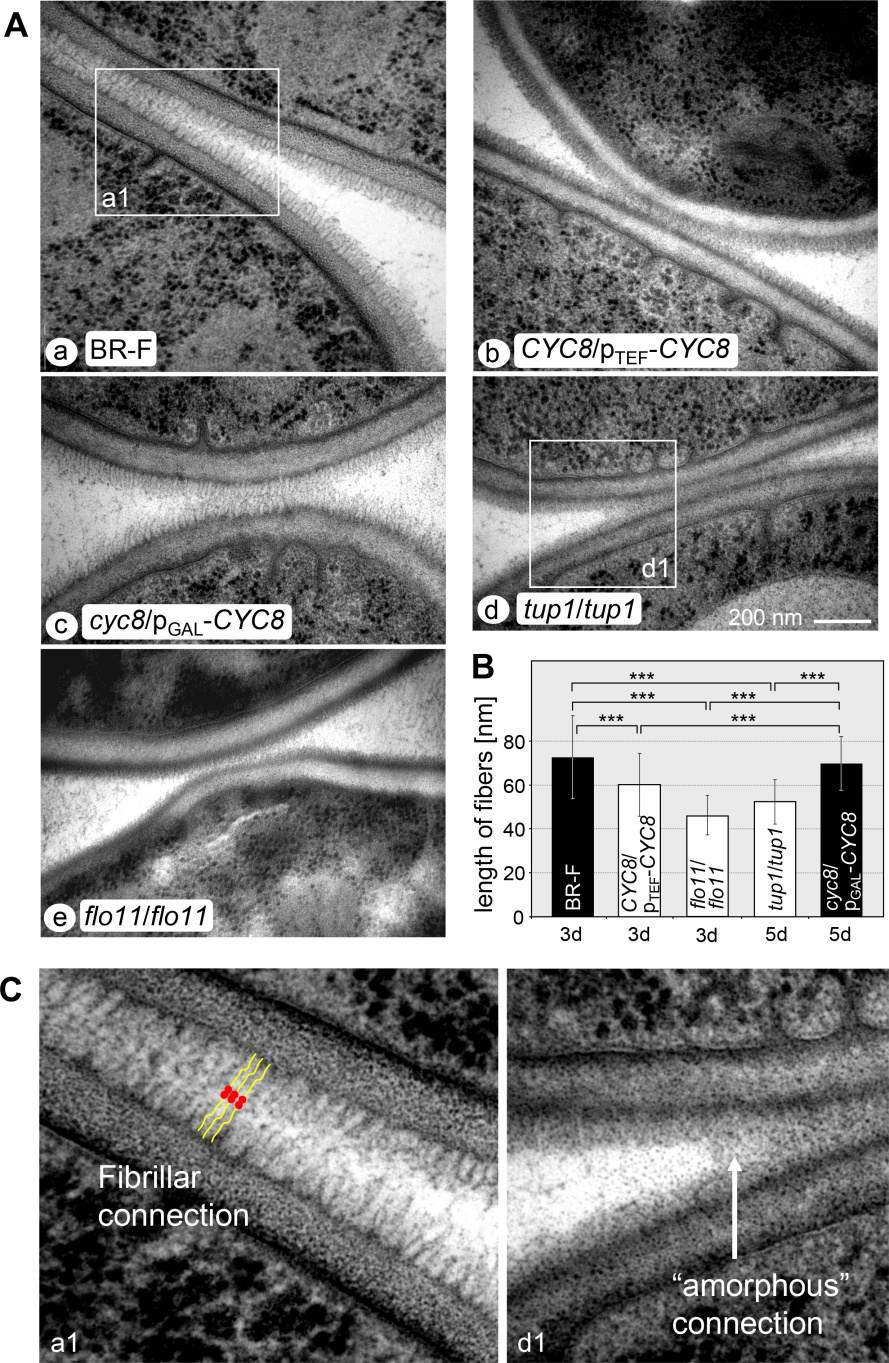
Extracellular fibrillar material between cells within colonies of strains with altered levels of Cyc8p and Tup1p. A, Electron microscopy of Velcro connections between cells from 3-day-old (a, b, e) and 5-day-old (c, d) colonies grown on GMA. B, The average length of the fibers is shown in the graph. Black columns, structured colony biofilms; white columns, smooth colonies; bars, SD; ***, P<0.001. C, Examples of extracellular material in higher magnification from colony biofilms (BR-F, inset a1 from Aa) and from smooth colonies (*tup1*, inset d1 from Ad). Velcro-like connections in BR-F are schematically indicated in yellow color, the red color indicates N-terminal Flo11A domains [48], potentially involved in the interaction.

**Fig 6:**
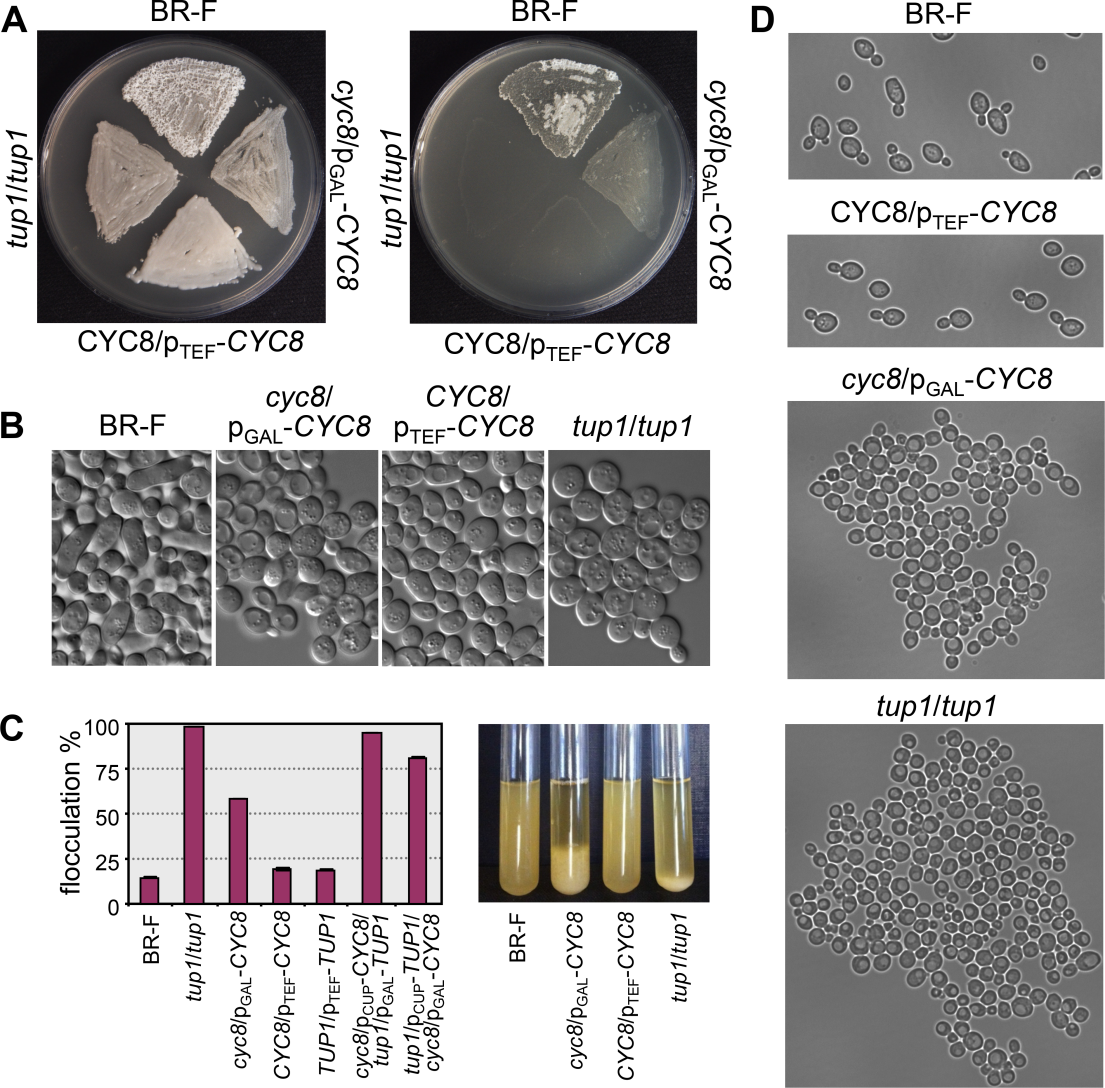
Cell morphology and adhesion characteristics within colonies of strains with altered levels of Cyc8p and Tup1p. A, Cell invasion (right picture) shown after washing the cells grown on GMA plates for 3 days (left picture). B, Morphology of cells from the aerial parts of 3-day-old colonies grown on GMA. C, Cell flocculation in liquid cultures grown for 2 days in GM (graph); examples of flocculation in tubes (right) and D, microscopic pictures of free cells and flocs.

Long fibers forming Velcro-like structures in contact sites between the cells were identified in colony biofilms, but not in smooth colonies of the BR-F-*flo11* strain, by transmission electron microscopy (TEM) of chemically fixed cells [6]. Here we used high-pressure freezing and freeze substitution TEM that improves identification of these structures and revealed some less abundant, extracellular fibrillar material even on the surface of cells within BR-F-*flo11* colonies. Fig 5A thus shows that cells in both structured and smooth colonies are covered on their surface by extracellular fibrillar material, which connects adjacent cells. However, the fibers in this material were significantly (20–30%) longer in the structured colony biofilms of the BR-F and non-induced p_GAL_-*CYC8* strains than in the smooth colonies of p_TEF_-*CYC8*, *tup1* and *flo11* strains (Fig 5B), in which shorter fibers are occasionally visible despite the material appearing to be less structured. The differences are evident also in cell-cell contact sites, where Velcro-like connections were visible among cells in colony biofilms, whereas less structured material was present at contact sites in smooth colonies (Fig 5C). Velcro-like connections may be caused by interaction of N-terminal Flo11A domains of Flo11p as reported in [48] (Fig 5C, indicated by red mark), although direct proof of the presence of Flo11p in these fibers is still lacking. Adhesion to solid surfaces and invasive growth are typical features of fungal biofilms [12] as well as of colony biofilms [13], which are evident particularly in the area of the colony roots [6]. Cell adhesion and agar invasion are absent in *S*. *cerevisiae flo11* colonies [10,13,49]. Figs 1 and 6A show that the p_TEF_-*CYC8* and *tup1* strains exhibited defects in invasive growth and adhered poorly to the agar. However, similar to the wt strain, cells of the non-induced p_GAL_-*CYC8* strain adhered to the agar even with robust washing. These data show that both organization of extracellular fibrillar material involved in cell-cell contact and cell adhesion to surfaces are antagonistically regulated by Cyc8p and Tup1p.

Adhesion to, and invasiveness into agar did not correlate with cell morphology. BR-F colony biofilms were formed by both oval and elongated cells in the aerial part and by pseudohyphae consisting of elongated cells in the subsurface part (Figs 1B and 6B). In contrast, there was a failure to form elongated cells by, not only smooth colony-forming strains with decreased level of Tup1p (*tup1*) and increased level of Cyc8p (p_TEF_-*CYC8*), but also a colony-biofilm forming strain with reduced level of Cyc8p (p_GAL_-*CYC8*). Thus, although the p_GAL_-*CYC8* strain formed (in the absence of galactose) a structured colony biofilm, its root part was formed by chains of rounded cells that divided by monopolar budding and invaded the agar (Fig 1B). Consistently, some wild *S*. *cerevisiae* strains form structured colony biofilms despite being unable to form typical pseudohyphae composed of elongated cells [13]. Tup1p and Ssn6p (Cyc8p) are repressors of invasive/filamentous growth in *C*. *albicans* [38,41,50]. Our data show that in *S*. *cerevisiae*, an imbalance in the Cyc8p and Tup1p levels, rather than the presence or absence of an individual regulator, diminishes cell elongation. This defect, however, does not influence the colony biofilm morphology.

### Cyc8p and Tup1p co-repress cell flocculation

The Cyc8p-Tup1p complex has been implicated in the repression of genes involved in cell flocculation, such as *FLO1* [17,51]. Consistent with the literature [52–54], either deletion of *TUP1* (*tup1* strain) or substantial reduction of *CYC8* expression (p_GAL_-*CYC8* without galactose) or reduction of *TUP1* and *CYC8* expression (p_CUP_-*CYC8*/p_GAL_-*TUP1* or p_CUP_-*TUP1*/p_GAL_-*CYC8* strain without inducing compound) resulted in the formation of macroscopic flocs (clusters of cells) that sedimented efficiently (Fig 6C and 6D), indicating de-repression of the flocculation genes. In striking contrast, the wt strain BR-F and the *CYC8*- or *TUP1*- over-expressing strains (p_TEF_-*CYC8* or p_TEF_-*TUP1* strains) did not form cell clusters. These data show that in contrast to the antagonistic functions of Cyc8p and Tup1p in processes involved in colony biofilm formation, Tup1p and Cyc8p in concert repress other flocculation genes such as *FLO1* in the BR-F strain. These results are in agreement with findings showing that i) in contrast to *FLO11*, the expression of other flocculation genes (*FLO1*, *FLO9* and *FLO10*) is equivalent in colony biofilms and in smooth domesticated colonies and ii) citrate buffer treatment and the presence of mannose in the medium (both of which eliminate the flocculation caused by Flo1p but not that of Flo11p) affect BR-F cell flocculation in liquid culture but not the adhesiveness of cells from BR-F colonies [43] and iii) *FLO1* and *FLO5* play roles in cell aggregation and flocculation, whereas *FLO11* expression promotes invasive growth and biofilm formation [55].

### Conclusions

Our findings highlight a previously unknown antagonistic function of Tup1p and Cyc8p in the regulation of complexity of yeast colony biofilms. While Tup1p is essential for the formation of colony biofilms, increased levels of Cyc8p prevent formation of colony biofilms leading to formation of smooth colonies similar to those of laboratory strains. The antagonistic functions of Tup1p and Cyc8p are specific to features typical of yeast biofilm life-style, such as cell invasiveness, adhesion to semisolid surfaces and cell-cell adhesion by cell-wall fibers. Properties important for other types of multicellularity, such as cell flocculation [51], are regulated differently, being repressed by both regulators. In accordance, deletion of genes *NRG1*, *MIG1* or *SFL1* for repressors recruiting the Cyc8p-Tup1p co-repressor complex to promoters [56–58], did not prevent Cyc8p-mediated repression of colony biofilm formation in p_TEF_-*CYC8* strain (S3 Fig).

Flo11p adhesin is key protein in colony biofilm formation affecting most of the above mentioned biofilm-specific processes [10,13,14]. We therefore tested a hypothesis that Cyc8p and Tup1p regulate biofilm-specific processes via regulation of Flo11p. Indications exist in previous research of a possible relationship between Cyc8p/Tup1p and *FLO11* expression, but findings were not consistent. Both positive [59–61] and negative [59] effects of *TUP1* deletion on *FLO11* mRNA levels have been reported and deletion of the *CYC8* gene has been shown to increase *FLO11* mRNA levels [56]. Our in depth analyses revealed that Tup1p and Cyc8p regulate the level of Flo11p adhesin in the opposite manner and at different steps in its expression (Fig 4). Firstly, Cyc8p itself represses *FLO11* gene transcription, whereas Tup1p counteracts Cyc8p function, thus contributing positively to *FLO11* expression. Efficiency of Cyc8p-based *FLO11* gene repression depends on the comparative levels of Cyc8p and Tup1p proteins and we hypothesize that Tup1p can balance the level of free Cyc8p by forming a Cyc8p-Tup1p complex, which apparently does not regulate biofilm specific processes, but can regulate other cellular properties such as expression of flocculins. Four molecules of Tup1p interact with one molecule of Cyc8p [26], which means that, in accordance with our data, a smaller change in Cyc8p than in Tup1p levels has a stronger effect on *FLO11* expression. Secondly, Tup1p also positively regulates the level of Flo11p protein in colony biofilms by preventing its degradation. Flo11p is targeted to the cell wall via the secretory pathway and is partially shed into the extracellular space [46]. The mechanism of its degradation and involvement of specific protease(s) are currently unknown. Our data showed that Tup1p prevents degradation of extracellular Flo11p-GFP. Tup1p thus may repress expression of a gene coding for a cell wall protease that is involved in Flo11p degradation. Interestingly, Tup1p represses a set of secreted aspartyl proteinases (SAPs) in *C*. *albicans* [62] and derepression of genes coding for extracellular proteases was observed in an *Aspergilus nidulans* strain deleted in the TUPA gene (an ortholog of *TUP1*) [63].

In summary, our study identifies Cyc8p and Tup1p as important regulators of Flo11p gene expression and protein stability, both affecting the final Flo11p amount in cells and the extracellular space of yeast multicellular structures. According to Flo11p concentration, the structures then acquire different levels of complexity ranging from smooth colonies to colony biofilms. Fine tuning of the amounts and mutual effects of Cyc8p and Tup1p regulators in colony/biofilm cell subpopulations could also provide a mechanism for balancing Flo11p levels and related cellular properties at different positions within the structure and, potentially, at different times during its development. In this respect, further studies are required to uncover potential role of Cyc8p and Tup1p in regulating the amount of Flo11p in different parts of colony biofilms, such as in the internal colony parts, where Flo11p seem not to be present. Orthologs of both Tup1p and Cyc8p are present in different yeast and other fungal species and their role in filamentation, phenotypic switching and virulence of yeast/fungal pathogens has been recently suggested [39–41,50,64]. Identification of Tup1p as an important positive regulator of the formation of colony biofilms brings practical advantages, making *TUP1* orthologs in pathogenic yeasts prospective gene targets for new antifungal treatment strategies.

## Materials and methods

### Yeast strains and media

All strains prepared in this study (Table 1) were derived from the wild yeast strain BR-F from a collection at the Institute of Chemistry (Slovak Academy of Sciences) and its derivative BR-F-Flo11p-GFP [15]. Colonies were grown on GMA (3% glycerol, 1% yeast extract, and 2% agar) at 28°C unless otherwise indicated, at densities ranging from 10^3^ to 6 x 10^3^ cells per plate. For the flocculation tests, the strains were grown in liquid GM (GMA without agar). For the strain constructions, G418 and nurseothricin concentrations in GMA were 200 and 100 μg/ml.

In galactose/Cu^2+^ induction experiments, colonies were grown 3 days on GMA. Then, the agar was supplemented by galactose and/or Cu^2+^ to a final concentrations of either 2% galactose and/or 3 mM CuSO_4_ for colony incubation for 4 h (for Northern blot and LC-MS/MS) or 0.1% galactose and/or 0.25 mM CuSO_4_ for longer 18 h incubations used for morphology experiments and determination of Flo11p-GFP levels by Western blots (lower concentrations of both galactose and Cu^2+^ were needed to avoid artificially affecting colony morphology in longer incubations).

### Colony imaging

Colony images were captured in incident and/or transmitted light. A ProgRes CT3 CMOS camera with a Navitar objective and NIS Elements software (Laboratory Imaging, s.r.o, Prague, CZ) were used.

### Strain constructs

Strains with gene deletions and with genes under the control of an artificial promoter (p_CUP_, p_GAL_ and p_TEF_) were prepared according to [65–67]; primers and plasmids are listed in S1 Table. Yeast cells were transformed as described in [68]. Correct genomic integration of cassettes was verified by PCR using specific primers and by sequencing. Cre-lox system was used to remove antibiotic resistance genes in strains subjected to multiple manipulations [66].

### 2PE-CM of microcolonies

The internal architecture of the microcolonies was visualized by two photon excitation confocal microscopy (2PE-CM) according to [6,69]. In brief, colonies were embedded in agarose and cut vertically down the middle. The cut surface was placed on a coverslip, and colony side views were obtained by 2PE-CM. When required, the cross-sections were stained with 1 μg/ml Calcofluor white. Excitation wavelengths of 920 nm and 790 nm and the emission bandwidths of 480–595 nm and 400–550 nm were used for GFP and Calcofluor white. An overview of the morphology of colonies was obtained simultaneously with green GFP fluorescence as autofluorescence in the 600-740-nm wavelength range. Images of the whole colonies (Figs 1A, 1B, 2C and 2D) and the central parts of the colonies (Fig 1B) were obtained by combining two or three images from neighboring fields of view.

### Determination of Flo11p-GFP levels by western blots and of Tup1p and Cyc8p by nanoLC-MS-MS

The detection of GFP tagged Flo11p (in cell lysates or the extracellular fluid) by western blots was performed as described [70]. In brief, cells harvested from colonies were broken by glass beads in the presence of protease inhibitors, and proteins (25 μg/lane) of cell lysates were subjected to SDS-PAGE. GFP was detected by mouse monoclonal horseradish peroxidase (HRP)-conjugated anti-GFP antibody (Santa Cruz). Membranes stained by Coomassie blue were used as loading controls (S4 Fig). Extracellular proteins were extracted by phosphate-saline buffer from 3-day-old colonies. After centrifugation, proteins of the supernatant were precipitated by methanol/chloroform treatment [71]. Extracellular proteins extracted from 50 mg of wet biomass were loaded to each slot.

For nanoLC-MS-MS analysis, the cells were disrupted in 100 mM triethylammonium bicarbonate buffer using glass beads. Protein aliquots (30 μg; determined by the bicinchoninic acid assay, Sigma) were solubilized using sodium deoxycholate (1% (w/v) final conc.), reduced with tris(2-carboxyethyl)phosphine, alkylated with S-methyl methanethiosulfonate, digested sequentially with trypsin and extracted with ethylacetate saturated with water [72]. Samples were desalted using C18 sorbent (Supelco pn: 66883-U) and eluents were dried and resuspended in 20 μl of 1% trifluoroacetic acid. Peptide (2 μg) from each sample were separated on 50-cm C18 column using 2.5 h elution gradient and analyzed in a DDA mode on Orbitrap Fusion Tribrid (Thermo Scientific) mass spectrometer. Three biological replicates were run for each strain and condition. Resulting raw files were processed in MaxQuant (v. 1.5.8.3) [73]. Searches were performed against latest version of *S*. *cerevisiae* Uniprot database and common contaminant database. Further analysis was performed in Perseus (v. 1.5.5.3) [74].

### Cell adherence and invasiveness

The cell-cell adherence (flocculation) assay was performed according to [75]. In brief: 2-day-old cell cultures grown in GM medium were harvested, flocculation disrupted by EDTA (pH 8, 50 mM final concentration) and OD_600_ of the cell suspension determined (reading A). Then, cells were washed twice by dH_2_O and suspended in 30 mM CaCl_2_. After 60 s, OD_600_ at upper layers of the cell suspension was measured (reading B). Flocculation (%) was calculated according to the formula: 100*(A-B)/A. Average of 4 independent measurements +/- SD is shown. The flocs or free cells were photographed using transmission light microscopy (Microscope DMR, Leica, Germany). In the invasive growth assay [76] cells were streaked onto standard GMA plates and grown at 28°C for 3 days. Plates were vigorously washed with water and photographed.

### Electron microscopy

Samples were prepared according to [77] with some modifications. Briefly, 3-day- and 5-day-old colonies were frozen in an EM PACT2 high-pressure freezer (Leica, Germany). The samples were freeze-substituted in an automatic FS machine (Leica, Germany) in 100% acetone containing 2% osmium tetroxide as follows: -90°C for 96 h, 5°C increase per hour for 14 h, -20°C for 24 h, 3°C increase per hour for 8 h, and 4°C for 18 h. The substituted samples were embedded in pure Epon. Ultrathin sections were cut using a Reichert-Jung Ultracut E ultramicrotome and stained using uranyl acetate and lead citrate. The sections were examined using a JEM-1011 transmission electron microscope (JEOL, Japan) operating at 80 kV. Fine structure measurements were performed using a Veleta camera and iTEM 5.1 software (Olympus Soft Imaging Solution GmbH).

### RNA isolation and northern blotting

Colonies were suspended in TES buffer (10 mM Tris, pH 7.5, 10 mM EDTA, 0.5% SDS) and total RNA isolated using the hot phenol method [78]. Fifteen micrograms of total RNA was denatured in loading buffer with formamide, separated in 1.5% agarose gel and transferred to a positively charged nylon membrane (Amersham Hybond-XL, GE Healthcare Ltd). The membranes were hybridized with specific DNA probes prepared using a random primer labeling kit (Takara). The rRNA content was visualized by ethidium bromide staining of gels and used as a loading control (S2 Fig).

## Supporting information

S1 Fig Level of *CYC8* mRNA and architecture of colony biofilms formed by *cyc8*/p_GAL_-*CYC8* strain. A, Northern blot (NB) showing level of *CYC8* mRNA in BR-F and *cyc8*/p_GAL_-*CYC8* colony biofilms (without galactose). B, Development of architecture of BR-F colony biofilm and more slowly growing *cyc8*/p_GAL_-*CYC8* colony biofilm.
(PDF)

S2 Fig Level of p_GAL_-regulated *CYC8* and *TUP1* mRNA in presence/absence of copper. (PDF)

S3 Fig Comparison of colonies of p_TEF_-*CYC8* strain and KO strains. (PDF)

S4 Fig Loading controls. Loading controls for western blots in Fig 2B (A) and Fig 3C (C) and for northern blots in Fig 3A (B).
(PDF)

S5 Fig Galactose does not affect BR-F colony morphology in the used set-up. A, 3-day-old BR-F colonies grown on GMA plates were treated by galactose (120 μl of 10% galactose) applied to the wells and cultivated for 18 h. B, Untreated BR-F colonies of the same age grown on GMA plates.
(PDF)

S1 Table Primers and Plasmids. (PDF)
