# Supplementary material for: Cyc8p and Tup1p transcription regulators antagonistically regulate Flo11p expression and complexity of yeast colony biofilms
Source: PLoS Genet. 2018 Jul 2;14(7):e1007495. doi: 10.1371/journal.pgen.1007495 (PMC6044549; doi:10.1371/journal.pgen.1007495)
Supplement: S1 Fig — A, Northern blot (NB) showing level of CYC8 mRNA in BR-F and cyc8/pGAL-CYC8 colony biofilms (without galactose). B, Development of architecture of BR-F colony biofilm and more slowly growing cyc8/pGAL-CYC8 colony biofilm. (PDF) [file pgen.1007495.s001.pdf]

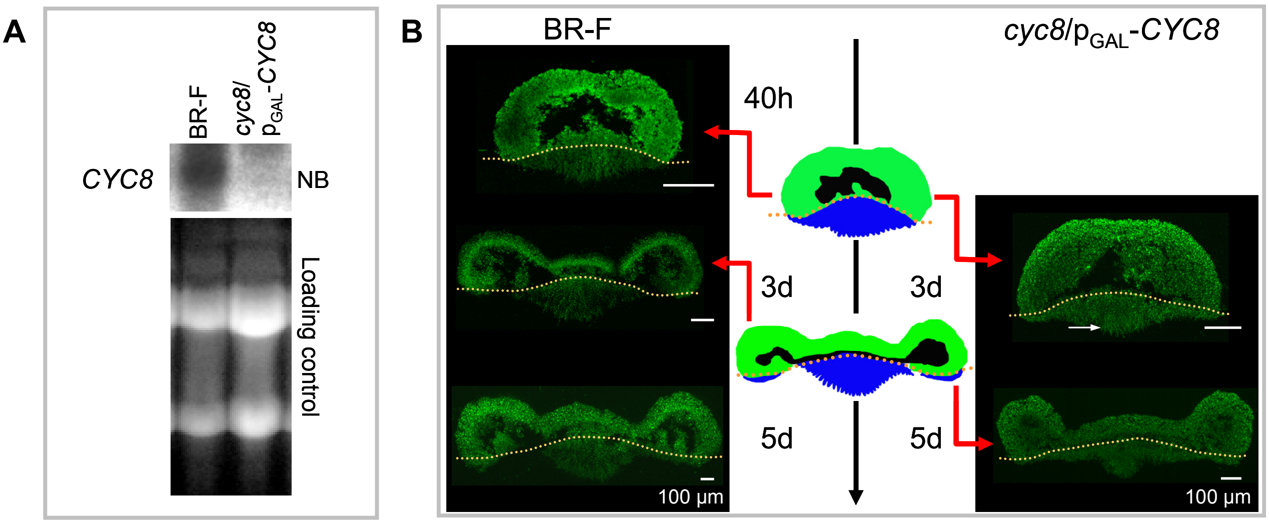

S1 Fig: Level of *CYC8* mRNA and architecture of colony biofilms formed by *cyc8*/p$_{GAL}$-*CYC8* strain

A, Northern blot (NB) showing level of *CYC8* mRNA in BR-F and *cyc8*/p$_{GAL}$-*CYC8* colony biofilms (without galactose). Loading control is below. B, Development of architecture of BR-F colony biofilm and more slowly growing *cyc8*/p$_{GAL}$-*CYC8* colony biofilm. Schemes of the two typical morphological forms are shown in the central section with typical features of colony biofilms marked: aerial part (in green), root invasive part (in blue) and cell-free cavity (in black). Yellow dotted line marks surface of the agar. Red arrows indicate the same morphological forms that are formed on different days of development of wild type (BR-F) and mutated (*cyc8*/p$_{GAL}$-*CYC8*) colony biofilms.
