# Supplementary material for: Cyc8p and Tup1p transcription regulators antagonistically regulate Flo11p expression and complexity of yeast colony biofilms
Source: PLoS Genet. 2018 Jul 2;14(7):e1007495. doi: 10.1371/journal.pgen.1007495 (PMC6044549; doi:10.1371/journal.pgen.1007495)
Supplement: S2 Fig — (PDF) [file pgen.1007495.s002.pdf]

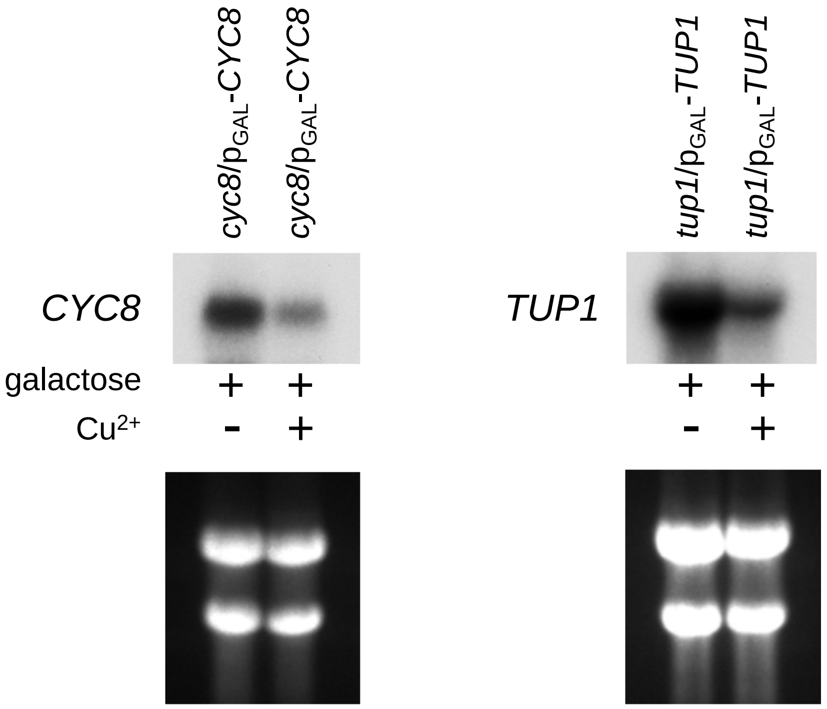

S2 Fig: Level of p_{GAL}-regulated *CYC8* and *TUP1* mRNA in presence/absence of copper.

p_{GAL}-*CYC8* and p_{GAL}-*TUP1* colonies were induced by galactose in presence or absence of copper. The same copper and galactose concentration and time of induction as in the Figure 3A were used.
