# Supplementary material for: Cyc8p and Tup1p transcription regulators antagonistically regulate Flo11p expression and complexity of yeast colony biofilms
Source: PLoS Genet. 2018 Jul 2;14(7):e1007495. doi: 10.1371/journal.pgen.1007495 (PMC6044549; doi:10.1371/journal.pgen.1007495)
Supplement: S3 Fig — (PDF) [file pgen.1007495.s003.pdf]

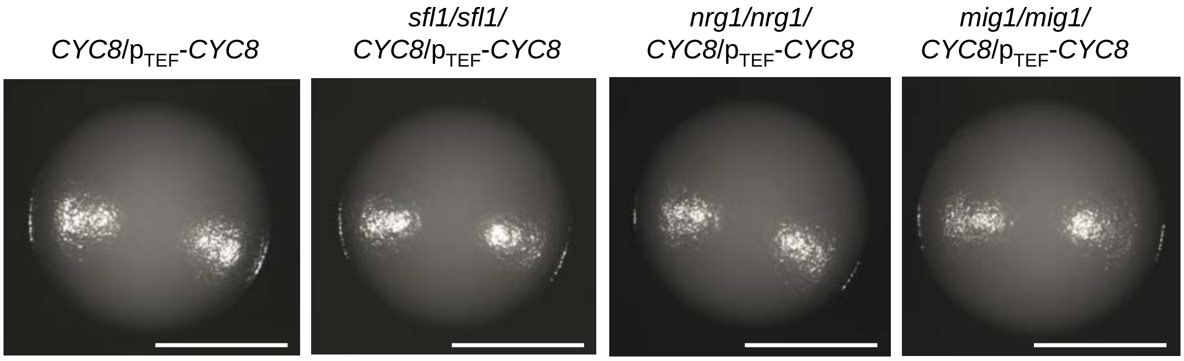

S3 Fig: Comparison of colonies of p$_{TEF}$-*CYC8* strain and KO strains. Strains with deletions of *SFL1*, *NRG1* and *MIG1* genes, respectively, were derived from p$_{TEF}$-*CYC8* strain. Colonies were grown 4 days on GMA. Bar, 1mm.
