# Supplementary material for: Cyc8p and Tup1p transcription regulators antagonistically regulate Flo11p expression and complexity of yeast colony biofilms
Source: PLoS Genet. 2018 Jul 2;14(7):e1007495. doi: 10.1371/journal.pgen.1007495 (PMC6044549; doi:10.1371/journal.pgen.1007495)
Supplement: S4 Fig — Loading controls for western blots in Fig 2B (A) and Fig 3C (C) and for northern blots in Fig 3A (B). (PDF) [file pgen.1007495.s004.pdf]

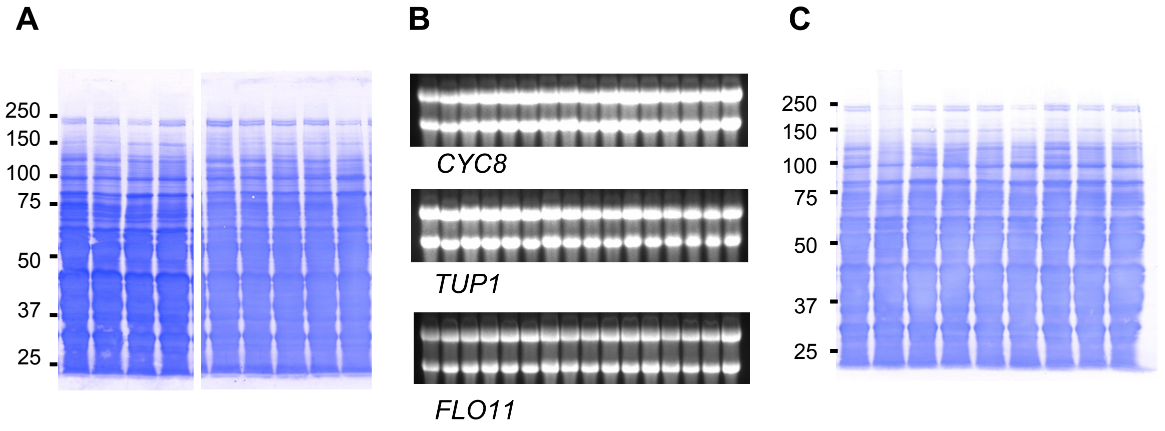

S4 Fig: Loading controls

Loading controls for western blots in Figure 2B (A) and Figure 3C (C) and for northern blots in Figure 3A (B).
