# Supplementary material for: Cyc8p and Tup1p transcription regulators antagonistically regulate Flo11p expression and complexity of yeast colony biofilms
Source: PLoS Genet. 2018 Jul 2;14(7):e1007495. doi: 10.1371/journal.pgen.1007495 (PMC6044549; doi:10.1371/journal.pgen.1007495)
Supplement: S5 Fig — A, 3-day-old BR-F colonies grown on GMA plates were treated by galactose (120 μl of 10% galactose) applied to the wells and cultivated for 18 h. B, Untreated BR-F colonies of the same age grown on GMA plates. (PDF) [file pgen.1007495.s005.pdf]

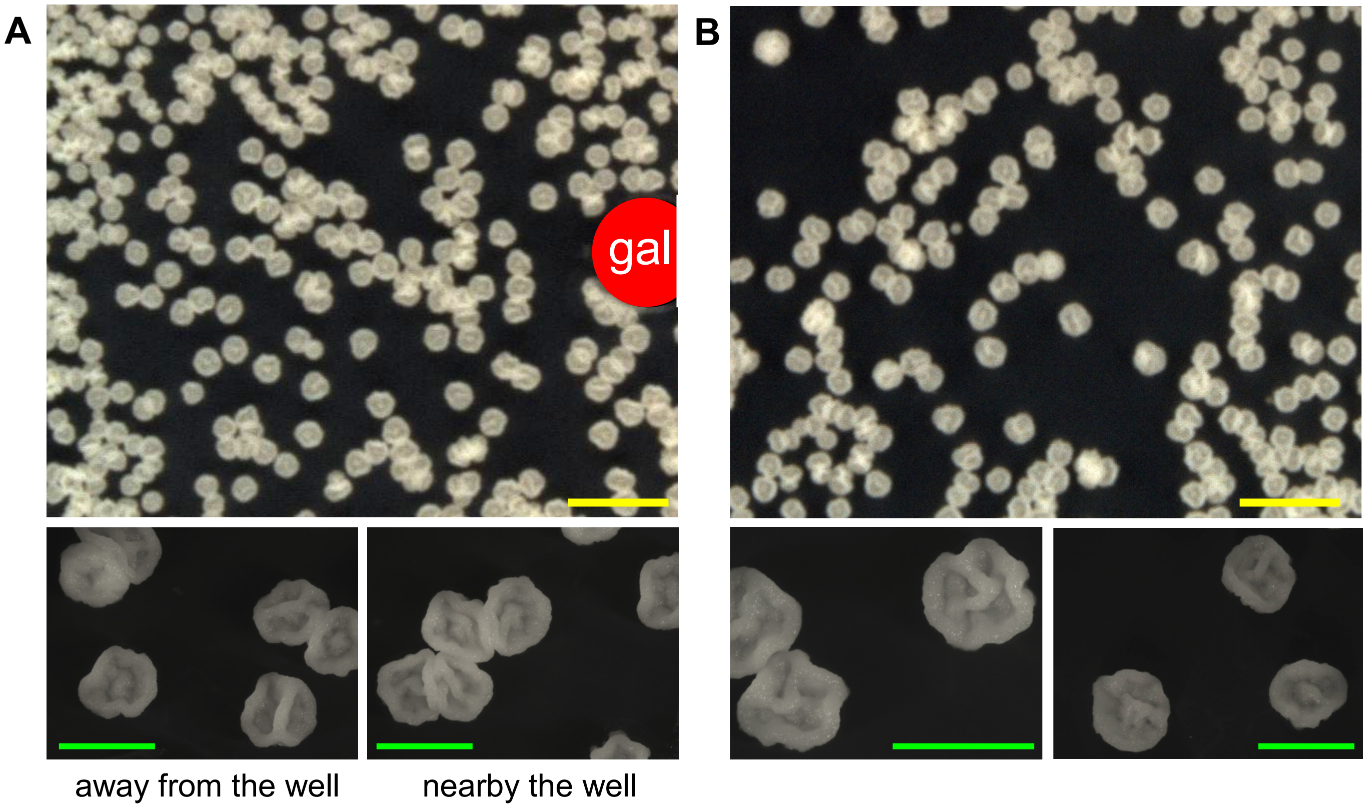

S5 Fig: Galactose does not affect BR-F colony morphology in the used set-up. A, 3-day-old BR-F colonies grown on GMA plates were treated by galactose (120 µl of 10% galactose) applied to the wells and cultivated for 18 h. B, Untreated BR-F colonies of the same age grown on GMA plates. Yellow bar, 5 mm; green bar, 2 mm.
