# Supplementary material for: Cyc8p and Tup1p transcription regulators antagonistically regulate Flo11p expression and complexity of yeast colony biofilms
Source: PLoS Genet. 2018 Jul 2;14(7):e1007495. doi: 10.1371/journal.pgen.1007495 (PMC6044549; doi:10.1371/journal.pgen.1007495)
Supplement: S1 Table — (PDF) [file pgen.1007495.s006.pdf]

Table S1. Primers and Plasmids

A. List of the primers.

**Primer**	**Sequence**	**Purpose**
CYC8-del-forward	AACAACAACAAACAAAACACGACTGGAAAAAAAAAAATTAGGAA<br>AACAGCTGAAGCTTCGTACGC	deletion of *CYC8* gene
CYC8-del-reverse	GATTATAAATTAGTAGATTAATTTTTTGAATGCAAACTTTGCATA<br>GGCCACTAGTGGATCTG	deletion of *CYC8* gene
TUP1-del-forward	TGATAAGCAGGGGAAGAAAGAAATCAGCTTTCCATCCAAACCAA<br>TCAGCTGAAGCTTCGTACGC	deletion of *TUP1* gene
TUP1-del-reverse	GTTTAGTTAGTTACATTTGTAAAGTGTTCCTTTTGTGTTCTGTTCG<br>CATAGGCCACTAGTGGATCTG	deletion of *TUP1* gene
GAL1_CYC8 forward	AACAACAAACAAAACACGACTGGAAAAAAAAAAATTAGGAAAAA<br>TGCGTACGCTGCAGGTCGAC	Insertion of p_{GAL} or p_{CUP} in front of *CYC8* coding sequence
GAL1_CYC8-reverse	CTGTTGAGCGGGTTGTTCCATTATTGTTTGTTCACCGCCCGGATT<br>CATCGATGAATTCTCTGTCG	Insertion of p_{GAL} or p_{CUP} in front of *CYC8* coding sequence
TEF1_CYC8 -forward	AACAACAAACAAAACACGACTGGAAAAAAAAAAATTAGGAAAAA<br>TGCGTACGCTGCAGGTCGAC	Insertion of p_{TEF} in front of *CYC8* coding sequence
TEF1_CYC8 -reverse	CTGTTGAGCGGGTTGTTCCATTATTGTTTGTTCACCGCCCGGATT<br>GGATCCACTAGTTCTAGA	Insertion of p_{TEF} in front of *CYC8* coding sequence
GAL1_TUP1 -forward	TAAGCAGGGGAAGAAAGAAATCAGCTTTCCATCCAAACCAATAT<br>GCGTACGCTGCAGGTCGAC	Insertion of p_{GAL} or p_{CUP} in front of *TUP1* coding sequence
GAL1_TUP1- reverse	GCTCATTCAGCTTATTCTGCGTATTCGAAACGCTGGCAGTCATCG<br>ATGAATTCTCTGTCG	Insertion of p_{GAL} or p_{CUP} in front of *TUP1* coding sequence
NRG1-del-forward	TTCCTCTCGACCAGCATATTACTACCCTTCGCAAACTTTCAGGCA<br>CAGCTGAAGCTTCGTACGC	deletion of *NRG1* gene
NRG1-del-reverse	CGGAATAGTAGTACTGCTAATGAGAAAAACACGGGTATACCGTC<br>AA GCATAGGCCACTAGTGGATCTG	deletion of *NRG1* gene
MIG1-del-forward	ACACGAGAGTTGAGTATAGTGGAGACGACATACTACCATAGCCC<br>AGCTGAAGCTTCGTACGC	deletion of *MIG1* gene
MIG1-del-reverse	CTATTGTCTTTTGATTTATCTGCACCGCCAAAAACTTGTCAGCGT<br>AGCATAGGCCACTAGTGGATCTG	deletion of *MIG1* gene
SFL1-del-forward	GGTCCTAAGACAGCACAAATCAGTTATATAGAAAAAAAGAAGA<br>AAAAATC CAGCTGAAGCTTCGTACGC	deletion of *SFL1* gene
SFL1-del-reverse	GAGGTGCTTTGAACTTTTAGACAACTAGAGATTAAAAAGGCAAA<br>GA GCATAGGCCACTAGTGGATCTG	deletion of *SFL1* gene

B. List of the plasmids.

**Plasmid**	**Application**	**Reference**
pAG25	deletion cassette (nat1 marker)	(Goldstein and McCusker, 1999)
pUG6	deletion cassette (KanMX marker)	(Gueldener et al., 2002)
pYM-N23	p_{GAL1} cassette	(Janke et al., 2004)
pYM-N20	p_{TEF1} cassette	(Janke et al., 2004)
pYM-N1	P_{CUP1} cassette	(Janke et al., 2004)
pSH69	Cre expression plasmid	(Hegemann and Heick, 2011)
pUG6-25	deletion cassette (nat1 marker, with Cre-loxP site)	Modified from pUG6
pUG6-32	Deletion cassette (HygR marker, with Cre-loxP site)	Modified from pUG6

References:

Goldstein, A.L., and J.H. McCusker. 1999. Three new dominant drug resistance cassettes for gene disruption in Saccharomyces cerevisiae. *Yeast (Chichester, England)*. 15:1541-1553.

Gueldener, U., J. Heinisch, G.J. Koehler, D. Voss, and J.H. Hegemann. 2002. A second set of loxP marker cassettes for Cre-mediated multiple gene knockouts in budding yeast. *Nucleic acids research*. 30:e23.

Hegemann, J.H., and S.B. Heick. 2011. Delete and repeat: a comprehensive toolkit for sequential gene knockout in the budding yeast Saccharomyces cerevisiae. *Methods in molecular biology*. 765:189-206.

Janke, C., M.M. Magiera, N. Rathfelder, C. Taxis, S. Reber, H. Maekawa, A. Moreno-Borchart, G. Doenges, E. Schwob, E. Schiebel, and M. Knop. 2004. A versatile toolbox for PCR-based tagging of yeast genes: new fluorescent proteins, more markers and promoter substitution cassettes. *Yeast (Chichester, England)*. 21:947-962.
